# Association between the e-healthy literacy and cancer prevention consciousness in rural China: cancer cognition acting as a mediator

**DOI:** 10.1186/s41256-025-00421-1

**Published:** 2025-07-09

**Authors:** Huifang Zhang, Xindan Zhang, Xingli Ma, Boyang Fan, Yingjie Wang, Ao Zhang, Wenning Sun, Haining Yu, Haipeng Wang

**Affiliations:** 1 Center for Health Management and Policy Research, School of Public Health, Cheeloo College of Medicine, Shandong University, 128# Wenhua Xi Rd 44, Jinan, 250012 Shandong China; 2 NHC Key Laboratory of Health Economics and Policy Research (Shandong University), 128# Wenhua Xi Rd 44, Jinan, 250012 Shandong China; 3 Shandong Cancer Hospital and Institute, Shandong First Medical University and Shandong Academy of Medical Sciences, 440# Jiyan Rd, Jinan, 250117 Shandong China

**Keywords:** Cancer cognition, Cancer prevention consciousness, E-health literacy, Mediation, Rural residents

## Abstract

**Background:**

Cancer prevention is a critical public health challenge in China, especially among rural residents. This study aimed to examine the mediating role of cancer cognition in the relationship between e-health literacy and cancer prevention consciousness.

**Methods:**

A multi-stage stratified random sampling method was used to recruit 486 rural residents from Shandong Province for a questionnaire survey. Data from 453 valid responses were analyzed using descriptive statistics, univariate and multiple linear regression. Path analysis was used to examine the mediating role of cancer cognition in the relationship between e-health literacy and cancer prevention consciousness.

**Results:**

The mean score of cancer prevention consciousness among rural residents in this study was 7.46 out of a maximum of 10. Regression analysis showed that e-health literacy (β = 0.146, *P* < 0.001) and cancer cognition (β = 0.150, *P* < 0.001) influenced cancer prevention consciousness. Gender and the perceived necessity of cancer-related knowledge were also influencing factors (*P* < 0.001). The direct effect value of e-health literacy on cancer prevention consciousness was 0.155, which accounted for 84.87% of the total effect. The indirect effect value through cancer cognition level is 0.028, accounting for 15.13% of the total effect.

**Conclusions:**

We found an above-average level of cancer prevention consciousness among the rural residents. E-health literacy can enhance the  consciousness among individuals by increasing their cancer cognition. Policymakers should leverage e-health technologies to strengthen residents’ capacity to understand cancer-related information, with culturally tailored interventions further supporting effective prevention and global health efforts.

## Introduction

Cancer remains a leading cause of death worldwide and has emerged as a major public health concern in the twenty-first century. According to the International Agency for Research on Cancer (IARC), approximately 20 million new cancer cases were diagnosed in 2022, with 9.7 million deaths attributed to cancer, accounting for one-sixth of global mortality [1]. The most common cancer types include lung, colorectal, breast (female), prostate, stomach, and liver cancers, with lung cancer being the most frequent and deadliest among men, and breast cancer leading in incidence and mortality among women [1, 2]. In China, cancer-related deaths rose by 21.6% from 2005 to 2020, with higher mortality in rural areas than in urban areas. Notably, China has a higher cancer mortality rate than many developed countries [3, 4]. This upward trend has resulted in an enormous global burden of disease.

Globally, 44.4% of cancer deaths are attributed to behavioral risk factors, including tobacco use, alcohol consumption, physical inactivity, and poor dietary habits [5–7]. Primary cancer prevention, focusing on reducing these risk behaviors, is considered one of the most cost-effective strategies for lowering cancer incidence [6, 7]. However, limited access to health care for rural residents, coupled with their higher prevalence of unhealthy behaviors, exacerbates the urban–rural disparities associated with cancer [8]. In addition, evidence suggests that fewer rural residents are applying digital health compared to urban residents, contributing to the persistent threat of health inequalities between urban and rural areas [9]. This highlights the need to focus on digital health literacy and cancer prevention behaviors among rural residents to reduce the overall burden of cancer.

Some studies have found that cancer cognition level among rural residents in China is inversely related to cancer risk, and that rural residents with higher levels of cancer cognition are likely to have a lower risk of developing cancer [4, 10]. In other words, enhancing cancer cognition can facilitate the translation of preventive intentions into tangible actions, thereby reducing the risk of developing cancer [4]. Cancer cognition level, considered part of cancer health literacy, is the extent to which residents know basic cancer knowledge, risk factors, preventive and control behaviors [11–13]. Therefore, residents can learn to understand the knowledge related to cancer-causing factors and behaviors, which can help develop their behavioral intention to prevent cancer. Additionally, it has been reported that increased consciousness of breast cancer prevention and control through improved e-health skills and knowledge [14].

E-health literacy is the ability of individuals to obtain, understand, and evaluate health information through online electronic media [15]. Previous studies have shown that e-literacy can help individuals to understand information about their illnesses, thus enabling effective management of their illnesses [16–18]. Research have also shown that individuals with higher social status have easier access to social resources and information channels and usually have higher e-Health literacy, which greatly facilitates health-promoting behaviors such as disease prevention and disease management [9, 19, 20]. As a result, individuals with higher e-health literacy are more likely to adopt healthy behaviors such as quitting smoking and drinking, and engaging in regular exercise  in their lives to prevent the occurrence of their own cancers.

Previous studies have focused on the relationship between cancer cognition and prevention consciousness, while others have focused on the association between e-Health literacy and cancer prevention consciousness. However, the interaction among these three variables remains unclear. Building on the analysis of Bandura’s social cognitive theory, individuals engage in social learning by observing cancer prevention information in e-Health resources and integrate relevant knowledge and behavioral strategies through cognitive modeling. The resulting self-efficacy enhances confidence in preventive actions and encourages individuals to adopt proactive cancer prevention behaviors [21–24]. Therefore, this study aims to explore the relationship between e-health literacy, cancer cognition and cancer prevention consciousness, and examine the key factors affecting cancer prevention consciousness, in order to provide reference for improving rural residents’ health literacy and promoting cancer prevention and control.

## Methods

### Study design and participants

With a population of over 101 million, Shandong is the third most economically developed province in China [25]. It is nationally representative in terms of its demographic structure, cultural characteristics and social lifestyle. A cross-sectional study was conducted from August to September 2023 in Shandong Province, China. A multi-stage stratified sampling method was used to recruit respondents. First, three cities were selected based on their geographic location and economic status. Second, one district/county was randomly selected from each city, and three subdistricts/townships were chosen from each district/county. Finally, three villages were randomly selected from each subdistrict or township, resulting in a total of 27 sampled villages. In each sampled village, 18 respondents were randomly selected for questionnaire survey.

The inclusion criteria were as follow: (1) Aged ≥ 18 years. (2) Able to communicate clearly. (3) Willing to participate in the survey and having provided written informed consent. Exclusion criteria were as follow: (1) Diagnosed with severe mental illness, unconsciousness, or significant communication impairments. (2) Unable to complete the survey independently or with minimal assistance. (3) Refusing to participate or withdrawing consent during the survey process.

### Data collection

Prior to data collection, investigators underwent standardized training to ensure familiarity with the study procedures and reduce potential biases. This training included unifying survey techniques, clarifying questionnaire completion standards, and ensuring consistent communication across all interviews. All participants provided their written informed consent to participate in this study. The study was reviewed and approved by the institutional review board of Shandong University School of Public Health. (#LL20221120) A total of 486 questionnaires were collected, of which 453 were valid, resulting in a 93.21% response rate.

### Measures

**General information questionnaire.** The general information questionnaire collected demographic sociological characteristics and cancer-related health information from participants. Socio-demographic characteristics included the following: gender (female, male), age (< 60, 60–75, > 75 years old), educational level (primary school and below, primary school, middle school and above), marital status (married, unmarried), employment status (farmer, non-farmer), annual household income (CNY) (< 10,000, 10,000–30,000, > 30,000). There was also information on four health-related variables. Self-reported health status was categorized as “healthy”, “generally healthy”, and “unhealthy”. Family history of cancer was categorized as “yes” and “no”. Residents’ perceptions of the necessity of knowledge about cancer in healthy states included “necessary”, “unnecessary” and “unknown”. Economic burden of cancer prevention and treatment was distinguished between serious and non-serious cases.

**Cancer prevention consciousness.** We measured cancer prevention consciousness using the first part (cancer prevention consciousness) of the Cancer Prevention Health Literacy Questionnaire developed by the National Cancer Center of China [26]. This section contains 10 items that measure related to cancer prevention consciousness in response to known risk or protective factors. These factors involved in the cancer consciousness questionnaire are applicable to rural residents. For example, participants were asked if they would quit or reduce smoking upon learning that not smoking reduces the risk of lung cancer (yes/no). Each item is scored dichotomously (yes = 1, no = 0), with total scores ranging from 0 to 10. Higher scores indicate stronger intentions to engage in cancer prevention behaviors. The questionnaire has a Cronbach’s alpha of 0.61 and has been validated and empirically tested by several experts for good reliability.

**E-health literacy scale.** E-Health literacy is measured using the e-Health Literacy Scale (e-HEALS) developed by Norman and Skinner, which assesses the population’s ability to apply the knowledge gained to address or resolve health issues [27]. The revised Chinese version of the scale consists of 8 items [28]. It can be divided into 3 dimensions: a test of the ability to find and apply online health information (5 items). a test of the ability to evaluate or differentiate between good and bad health information (2 items). and a test of the ability to make decisions based on online health information (1 item). Each item was rated with a 5-point Likert scale from 1 (strongly disagree) to 5 (strongly agree). The scale’s total score ranged from 8 to 40, and was divided into three levels: low, medium and high (≤ 13, 13–26, ≥ 27). The Cronbach alpha for this scale in this study was 0.94, indicating good reliability.

**Cancer cognition level.** The cancer cognition was measured using the first part of the Cancer Health Literacy Scale developed by Chinese scholar Liu Jinhui [29]. The scale reflects the respondents’ understanding of cancer-related knowledge, including basic understanding of cancer, related clinical manifestations, risk factors, etc. The scale had 17 five-point Likert scale questions with these options (strongly disagree, disagree, moderate, agree, and strongly agree), and was successively assigned points of 1, 2, 3, 4, and 5. The sum of each item score was normalized, and the score was divided into three levels: low, medium and high (≤ 50, 50–80, > 80). The Cronbach alpha for this scale in this study was 0.97, indicating good reliability.

### Statistical analysis

IBM SPSS Statistics 26.0 was used for data analysis. Firstly, participants’ socio-demographic and cancer-related characteristics were summarized using frequency (N) and percentage (%), while cancer prevention consciousness scores were reported as mean and standard deviation (SD). Secondly, independent t-tests and one-way analyses of variance (ANOVA) were used to investigate the associations between these characteristics and cancer prevention consciousness. Thirdly, multiple linear regression analysis was performed to explore the intention factors of cancer prevention consciousness. Finally, bootstrap mediation analyses were conducted using a regression-based path analysis approach. In the mediation analysis, we treated the total score quintiles for e-health literacy and cancer cognition to narrow the numerical differences and analyzed them as continuous variables. The mediation model included 5,000 bootstrap samples to estimate confidence intervals for the indirect effects. An indirect effect was deemed statistically significant if its 95% confidence interval excluded zero. Statistical significance was inferred when *P* < 0.05.

## Results

### Basic characteristics and cancer prevention consciousness of participants

Table 1 summarizes the basic characteristics and distribution of cancer prevention consciousness among the 453 respondents. The sample was predominantly female (66.9%), married (83.0%), and working in agriculture (80.4%). Among the respondents, 72.0% had an annual family income below CNY 30,000, 58.3% reported a primary school education level or less, 60.3% rated their health as good, and 81.2% had no family history of cancer. Nearly half of the respondents (48.8%) believed that it is necessary to learn knowledge about cancer while in good health. About 76.6% of the participants believed that cancer prevention and control could cause serious economic burden. The majority of participants had moderate levels of e-health literacy (55.0%) and cancer cognition (66.0%).

**Table 1:** The basic characteristics and distribution of cancer prevention consciousness among participants (n = 453)

	n(%)	Cancer prevention consciousness (Mean ± SD)	*F/t*	P
*Gender*			− 5.159	0.000***
Male	150 (33.1%)	6.78 ± 2.09		
Female	303 (66.9%)	7.80 ± 1.92		
*Age*			38.119	0.000***
< 60	122 (26.9%)	8.67 ± 1.53		
60–75	240 (53.0%)	7.19 ± 1.98		
> 75	91 (20.1%)	6.56 ± 2.05		
*Education Level*			14.789	0.000***
Primary school and below	126 (27.8%)	6.95 ± 1.98		
Primary school	138 (30.5%)	7.12 ± 2.00		
Middle school and above	189 (41.7%)	8.05 ± 1.95		
*Marital status*			− 3.760	0.000***
Married	376 (83.0%)	7.63 ± 1.96		
Unmarried	77 (17.0%)	6.62 ± 2.18		
*Employment status*			− 4.211	0.000***
Farmer	364 (80.4%)	7.29 ± 2.07		
Non-farmer	89 (19.6%)	8.18 ± 1.72		
*Household income (CNY)*			34.655	0.000***
< 10,000	193 (42.6%)	6.67 ± 2.13		
10,000–30,000	133 (29.4%)	7.68 ± 1.85		
> 30,000	127 (28.0%)	8.44 ± 1.54		
*Self-reported health*			5.054	0.007**
Healthy	273 (60.3%)	7.60 ± 1.96		
Generally healthy	130 (28.7%)	7.48 ± 2.02		
Unhealthy	50 (11.0%)	6.62 ± 2.94		
*Family history of cancer*			2.066	0.039*
Yes	85 (18.8%)	7.87 ± 1.88		
No	368 (81.2%)	7.37 ± 2.06		
*The necessity of knowledge about cancer*			80.275	0.000***
Necessary	221 (48.8%)	8.43 ± 1.46		
Unnecessary	121 (26.7%)	7.12 ± 1.83		
Unknown	111 (24.5%)	5.90 ± 2.15		
*Economic burden of cancer prevention and treatment*			2.415	0.017*
Serious	347 (76.6%)	7.61 ± 1.89		
Non-serious	106 (23.4%)	6.99 ± 2.40		
*E-Health literacy*			50.981	0.000***
Low	82 (18.1%)	5.91 ± 2.34		
Medium	249 (55.0%)	7.43 ± 1.72		
High	122 (26.9%)	8.57 ± 1.70		
*Cancer cognition*			28.241	0.000***
Low	135 (29.8%)	6.44 ± 2.14		
Medium	299 (66.0%)	7.85 ± 1.83		
High	19 (4.2%)	8.58 ± 1.74		

*SD* standard deviation

F, refers to the variance value in one-way ANOVA. t, refers to the difference in means between two groups in an independent samples t-test. *p < 0.05, **p < 0.01, ***p < 0.001

By comparison of the means using independent t-test and ANOVA, the respondents of higher e-Health literacy and cancer cognition level presented higher cancer prevention consciousness scores (F = 50.981, *P* < 0.05; F = 28.241, *P* < 0.05). Significant differences were observed across various social-demographic and cancer-related factors, including gender, age, education level, marital status, employment status, household income, self-reported health status, family history of cancer, perceived necessity of knowledge about cancer, and the economic burden of cancer prevention and treatment (all *P* < 0.05).

### Multiple linear regression analysis of cancer prevention consciousness

Multiple linear regression was used to analyze the factors associated with cancer prevention consciousness, as presented in Table 2. In Model A, the association between e-Health literacy and cancer prevention consciousness was analyzed without considering cancer cognition. The results indicated that both medium e-Health literacy (β = 0.157, *P* < 0.001) and high e-Health literacy (β = 0.233, *P* < 0.001) were positively associated with cancer prevention consciousness. In Model B, after including cancer cognition level as an additional variable, medium e-Health literacy (β = 0.146, *P* < 0.001), high e-Health literacy (β = 0.216, *P* < 0.001), and medium cancer cognition level (β = 0.150, *P* < 0.001) all remained significantly associated with cancer prevention consciousness. The reduction in the coefficients for e-Health literacy suggests a mediating effect of cancer cognition. Additionally, gender and the perceived necessity of cancer-related knowledge were also significant factors. (*P* < 0.001)

**Table 2:** Multiple linear regression analysis of factors influencing cancer prevention consciousness

	Model A	Model B
*Gender (Ref.* = *Male)*		
Female	0.263 (0.793, 1.478)***	0.265 (0.804, 1.482)***
*Age (Ref.* = *Age* < *60)*		
60–75	− 0.083 (− 0.772, 0.098)	− 0.080 (− 0.755, 0.107)
> 75	− 0.100 (− 1.092, 0.081)	− 0.078 (− 0.981, 0.188)
*Education level (Ref.* = *Education level* < *Primary school)*		
Primary school	− 0.025 (− 0.509, 0.292)	− 0.060 (− 0.673, 0.143)
≥ Middle school	0.035 (− 0.322, 0.614)	− 0.013 (− 0.530, 0.426)
*Marital status (Ref.* = *Married)*		
Not Married	− 0.062 (− 0.774, 0.102)	− 0.048 (− 0.697, 0.175)
*Employment status (Ref.* = *Farmer)*		
Non-farmer	− 0.017 (− 0.521, 0.349)	− 0.020 (− 0.533, 0.334)
*Household income (CNY) (Ref.* = *CNY* < *10,000)*		
10,000–30,000	0.052 (− 0.164, 0.624)	0.057 (− 0.137, 0.644)
> 30,000	0.097 (− 0.023, 0.901)	0.091 (− 0.045, 0.871)
*Self-reported health (Ref.* = *Healthy)*		
Generally healthy	0.016 (− 0.276, 0.418)	0.013 (− 0.289, 0.401)
Unhealthy	− 0.072 (− 0.974, 0.040)	− 0.072 (− 0.971, 0.034)
*Family history of cancer (Ref.* = *Yes)*		
No	− 0.006 (− 0.147, 0.883)	− 0.002 (− 0.399, 0.380)
*The necessity of knowledge about cancer (Ref.* = *Necessary)*		
Not necessary	− 0.178 (− 1.207, − 0.431)***	− 0.141 (− 1.046, − 0.251)***
Unknown	− 0.383 (− 2.226, − 1.388)***	− 0.361 (− 2.128, − 1.286)***
*Economic burden of cancer prevention and treatment (Ref.* = *Serious)*		
Not serious	0.022 (− 0.257, 0.468)	0.014 (− 0.291, 0.428)
*E-Health literacy (Ref.* = *Low)*		
Medium	0.157 (0.206, 1.027)***	0.146 (0.168, 1.027)***
High	0.233 (0.490, 1.643)***	0.216 (0.416, 1.566)***
*Cancer cognition (Ref.* = *Low)*		
Medium	–	0.150 (0.255, 1.030)***
High	–	0.079 (− 0.053, 1.648)
*Constant*	6.101 (4.599, 7.604)***	5.649 (4.133, 7.165)***
R2	0.417	0.014

*Ref* Reference

Model A was linear regression analysis for cancer prevention consciousness by not taking into account the variable “cancer cognition level”. Model B was linear regression analysis for cancer prevention consciousness by taking into account the variable “cancer cognition level”.*P < 0.05; **P < 0.01; ***P < 0.001

### The mediation effect of cancer cognition on the relationship between e-health literacy and cancer prevention consciousness

**Fig. 1:**
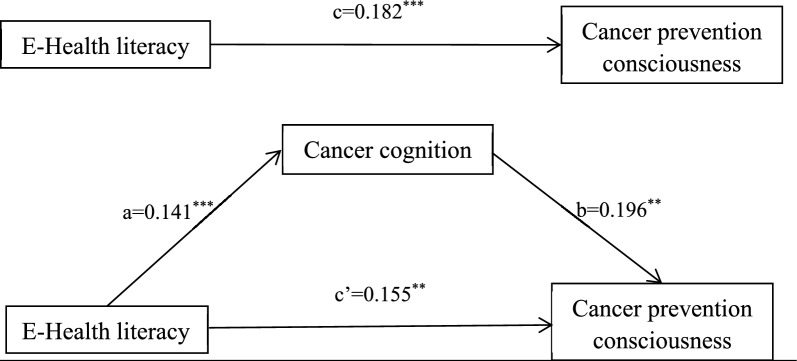
Mediating model of cancer cognition on the relationship between e-Health literacy and cancer prevention consciousness

After controlling the demographic variables (age, gender, education level, marital status, employment status, annual household income and self-reported health), we tested the mediating effect of cancer cognition on the relationship between e-health literacy and cancer prevention consciousness. (Table 3) The results showed 95% CI (0.060, 0.250) for the direct effect and 95% CI (0.005, 0.057) for the indirect effect, did not include zero, which confirmed the significance of the indirect effect of e-health literacy through cancer cognition level. In addition, the direct effect of e-Health literacy was 0.155, which accounted for 84.87% of the total effect. The indirect effect of e-Health literacy on the cancer prevention consciousness through the mediating  variable cancer cognition level was 0.028, representing 15.13% of the total effect. Figure 1 illustrated the mediation model, along with standardized path coefficients.

**Table 3:** Mediating effect of cancer cognition on the relationship between e-health literacy and cancer prevention consciousness

	Effect	SE	Bootstrap 95%CI	Relative effect value
Direct effect (c’)	0.155	0.048	0.060–0.250	84.87%
Indirect effect (a*b)	0.028	0.013	0.005–0.057	15.13%
Total effect (c)	0.182	0.048	0.089–0.276	100.00%

## Discussion

This study was designed to assess and enhance cancer prevention consciousness among rural residents. Our results revealed that the mean score for cancer prevention consciousness among rural residents was 7.46 out of a maximum of 10, suggesting a above-average level relative to the midpoint of the total score. While they demonstrate positive willingness and potential for improvement, further efforts are needed to enhance their preventive consciousness and behaviors. Previous studies have revealed that cancer prevention consciousness is poorer in the general population compared to cancer patients, especially among residents with lower literacy levels and incomes [30]. Several countries have effectively increased cervical cancer screening coverage in rural areas through educational interventions [31]. Therefore, there is a need for targeted health education for rural populations to raise consciousness of cancer prevention.

As expected, individuals with high e-health literacy had better cancer cognition and preventive consciousness, which is consistent with findings suggesting that e-health literacy has a positive impact on self-management in individuals with chronic diseases [17, 20]. Individuals with high e-health literacy typically have greater ability to retrieve, understand and apply information. They are more likely to proactively use e-health resources, such as medical guides and online courses, to acquire rich knowledge on cancer prevention, and thus develop better cancer cognition and cancer prevention behaviors [14, 32]. Low e-health literacy groups are more likely to miss out on cancer prevention resources because of barriers to information access, a phenomenon that exists among digitally disadvantaged groups in developing countries. Therefore, the application of e-health technologies in cancer prevention can be emphasized, and the sustainable enhancement of the digital health capacity of the entire population can be gradually realized through the development of digital health literacy assessments for citizens, including the ability to search for health information and the use of online counselling tools [21].

Previous studies have shown that knowledge of cancer risk factors directly influences the implementation of primary cancer prevention [33]. This result is validated by the finding that cancer cognition are positively associated with cancer prevention consciousness. On the one hand, when residents are well aware of the risk factors and early symptoms of cancer, they are more likely to take proactive preventive measures, such as regular health check-ups and changes in bad lifestyle habits. On the other hand, residents who had acquired cancer-related knowledge were better equipped to recognize similar early symptoms with greater confidence, enabling timely detection and intervention for potential health issues [34, 35]. This study suggests that community-based education can improve cancer screening behavior among Asian Americans [34]. Therefore, health education within villages and communities should be actively encouraged. Through easy-to-understand language and multiple forms of communication, knowledge of cancer risk and prevention can be effectively disseminated, thereby raising public awareness and fostering proactive health behaviors.

Our study found that cancer cognition mediated the relationship between e-Health literacy and cancer prevention consciousness. This is because the promotion of cancer prevention consciousness through e-health resources is influenced by the quality of cancer-related information, and high-level cancer cognition can address the issue [36]. However, the mediating effect was relatively weak. On the one hand, the better information discrimination and health service utilization skills of higher e-Health literacy groups can exist independently of cancer cognition [37]. On the other hand, the translation of cancer knowledge among rural residents is affected by factors such as barriers to accessing and operating digital devices, weakening the mediating effect [9]. In addition, persistent reliance on village doctors and cultural avoidance of cancer, which is known as an “unknown disease” in rural areas, weakening the efficiency of the conversion of e-health literacy to cancer cognition [38]. A mobile platform for educational resources with a dialect section can be developed, and the training and guidance of primary care physicians can help residents learn to use electronic devices, thereby improving the efficiency of translating cancer cognition among residents [39].

In addition, this study found significant differences in the distribution of cancer prevention consciousness among residents with different characteristics. Older farmers with lower income levels and limited educational attainment have poorer cancer prevention consciousness. The main reason for this is that these older adults have a limited level of cognition themselves and their children often live elsewhere, which limits their access to knowledge about cancer risk factors [40]. Moreover, this group of adults primarily addresses their financial needs and lack time to focus on their bad habits [41]. The study also found that residents with a family history of cancer, better self-rated health and those who felt that knowledge about cancer was necessary showed higher preventive consciousness. The possible reason is that they have higher health concerns and expectations, which stimulates their awareness of health protection and makes them more proactive in implementing preventive measures [10, 40]. Therefore, governments should emphasize differences in cancer prevention consciousness among populations. Health education should be targeted according to the economic status and health needs of the population to motivate key populations to adopt healthy behaviors for cancer prevention.

There are several limitations to this study. First, while the cross-sectional design validated the mediating effect of cancer cognition between e-Health literacy and prevention consciousness, the simultaneous measurement of variables resulted in the inability to rule out bidirectional causality. Despite this limitation, this study aims to explore the potential mechanisms of association between the variables, and therefore the cross-sectional design is still justified in the initial validation phase of the theoretical model. Second, the sample was limited to Shandong Province, which has geographical limitations and voluntary participation bias, limiting the generalizability of the findings to other regions of China. Third, the lack of pre-differentiation of cancer status may affect the generalizability of the results. In the future, longitudinal and intervention studies will be conducted to strengthen causal inferences. Recruitment of larger and broader samples, with stratified representation of healthy and affected populations, will increase the generalizability of the findings.

## Conclusions

The study indicates that the level of cancer prevention consciousness among rural residents in Shandong Province is above-average relative to the midpoint of the total score. E-health literacy not only contributes to the improvement of individual cancer perceptions and preventive consciousness, but also indirectly promotes the formation of positive cancer preventive behavioral intentions through the partial mediation of cancer cognition. These findings provide valuable guidance to improve cancer prevention behaviors among rural residents and thus promote global health. Therefore, governments should conduct targeted community-based cancer health education and e-health technology training to improve the ability of rural residents to understand and evaluate cancer health information online, thereby promoting a strong sense of cancer prevention.

## Data Availability

The datasets used and/or analyzed during the current study are available from the corresponding author on reasonable request.
